# An In Vitro Feline Macrophage Model as a Tool to Study Leishmania Infection and Glucuronoxylomannan-Mediated Modulation

**DOI:** 10.3390/biology15201792

**Published:** 2026-10-09

**Authors:** Fernanda de Paula Pepino, Ana Carolina Figueirinha, Renata Quintanilha dos Santos, Joyce de Meyrelles Borges, Elias Barbosa da Silva-Junior, José Osvaldo Previato, Lucia Mendonça-Previato, Danielle de Oliveira Nascimento, Celio Geraldo Freire-de-Lima, Debora Decote-Ricardo, Lucia Helena Pinto-da-Silva

**Affiliations:** 1 Departamento de Microbiologia e Imunologia Veterinária, Instituto de Veterinaria, Universidade Federal Rural do Rio de Janeiro, Seropédica 23897-000, RJ, Brazil; fernandapepino@ufrrj.br (F.d.P.P.); anacfgomes@ufrrj.br (A.C.F.); renataquintanilha@ufrrj.br (R.Q.d.S.); joyce@biof.ufrj.br (J.d.M.B.); daniongabi@gmail.com (D.d.O.N.); decotericardo@ufrrj.br (D.D.-R.); 2 Instituto de Biofísica Carlos Chagas Filho, Universidade Federal do Rio de Janeiro, Rio de Janeiro 21941-902, RJ, Brazil; eliasbarbosa@biof.ufrj.br (E.B.d.S.-J.); previato@biof.ufrj.br (J.O.P.); luciamp@biof.ufrj.br (L.M.-P.); celio@biof.ufrj.br (C.G.F.-d.-L.); 3 Instituto Nacional de Ciência e Tecnologia em Doenças Infecciosas e Crônicas das Mucosas e da Pele (INCT Mucosas e Pele), Universidade Federal de Minas Gerais, Belo Horizonte 31270-901, MG, Brazil

**Keywords:** Fcwf-4 feline macrophages, *Leishmania*, immunomodulation, purified capsular polysaccharide, glucuronoxylomannan (GXM)

## Abstract

Feline leishmaniasis is an emerging zoonotic disease, yet the cellular and immunological mechanisms underlying *Leishmania* infection in cats remain poorly understood. In this study, we established and characterized Fcwf-4 feline macrophages as an in vitro model of *Leishmania amazonensis* infection and investigated the immunomodulatory effects of glucuronoxylomannan (GXM), the major capsular polysaccharide of *Cryptococcus neoformans*. Fcwf-4 macrophages were infected with *L. amazonensis* promastigotes and evaluated at 2, 24, and 48 h post-infection. Parasite burden, macrophage viability, nitric oxide (NO), and reactive oxygen species (ROS) production were assessed in the presence or absence of GXM. Fcwf-4 macrophages supported *L. amazonensis* infection throughout the experimental period, with a progressive decrease in the infection index, indicating partial control of parasite burden over time. Notably, the presence of GXM increased the infection index without compromising macrophage viability, suggesting that enhanced parasite persistence resulted from immunomodulation rather than cytotoxicity. LPS stimulation enhanced NO and ROS production, whereas *L. amazonensis* infection selectively reduced NO production, indicating impairment of macrophage effector responses. Further, GXM suppressed ROS production, including basal ROS levels, suggesting an additional inhibitory effect on macrophage oxidative responses. Collectively, these findings provide experimental evidence that Fcwf-4 could be useful as a tool to study feline macrophage–*Leishmania* interactions in vitro and co-infection-associated immunomodulation.

## Simple Summary

Feline leishmaniasis is a disease caused by a parasite that can infect cats. However, little is known about how the cat’s immune system responds to this infection. In this study, we used a macrophage feline lineage to investigate how the parasite *Leishmania amazonensis* interacts with these cells. We also examined the effects of glucuronoxylomannan (GXM), a capsular polysaccharide produced by the *Cryptococcus neoformans*, which can alter immune responses. We found that feline macrophages were able to become infected with *L. amazonensis* and partially control the parasite over time. However, when GXM was present, more parasites survived inside the immune cells. This effect was not caused by damage or death of the macrophages, but rather by changes in their immune activity. *Leishmania* inhibited NO production and GXM also reduced the production of molecules involved in the cellular natural defenses against infections. These findings show a useful laboratory model for studying feline leishmaniasis and suggest that interactions between fungal and parasitic infections may influence disease progression.

## 1. Introduction

Leishmaniasis comprises a group of neglected zoonotic diseases distributed worldwide across more than 90 countries. Brazil is among the countries most affected by both cutaneous and visceral forms of the disease, with substantial impacts on human and animal health. Dogs are recognized as the main periurban reservoir of *Leishmania* parasites. Nevertheless, increasing evidence indicates a growing prevalence of *Leishmania* infection among domestic cats. In Brazil, studies have reported feline infection rates ranging from 6% to 30% in endemic areas, reaching 49% in some regions, such as Araçatuba, São Paulo State [1,2].

Over the past 40 years, approximately 80 cross-sectional studies investigating the prevalence of feline Leishmaniasis (FeL) worldwide have reported an average seroprevalence of 12% [3]. To date, six *Leishmania* species belonging to the subgenus *Leishmania* and one species belonging to the subgenus Viannia have been identified in domestic cats using molecular or isoenzyme-based typing methods [4]. Furthermore, the ability of *Leishmania* parasites to infect sand fly vectors during blood feeding on cats has been demonstrated [5]. Collectively, these findings support the hypothesis that domestic cats may act as an emerging reservoir host for *Leishmania*.

Coinfections with other microorganisms have also been reported in epidemiological studies of cats with leishmaniasis, including *Toxoplasma gondii*, *Sporothrix* spp., *Cryptococcus* spp., feline coronavirus, and, particularly, feline immunodeficiency virus (FIV) and feline leukemia virus (FeLV) [6,7,8,9,10]. In addition to coinfections, exposure to products derived from other microorganisms may modulate host susceptibility, immune responses, and disease progression, potentially contributing to the establishment and persistence of *Leishmania* infection in cats.

The immunological mechanisms underlying *Leishmania* infection in cats remain poorly understood, particularly those involving feline macrophages and their interactions with *Leishmania* parasites. Recently, feline monocyte-derived macrophages infected with *L. infantum* were shown to upregulate the expression of Th1-associated cytokines during in vitro infection [11]. Similarly, naturally infected cats exhibited higher serum concentrations of IL-12 and IFN-γ than uninfected animals [12]. These findings suggest that feline macrophages may mount a distinct immune response to *Leishmania* infection. However, the cellular and molecular mechanisms governing macrophage responses to *Leishmania* spp. remain largely unexplored, particularly in the context of coinfections or exposure to microbial products that may modulate parasite survival and host inflammatory responses.

The Fcwf-4 cell line, derived from feline (*Felis catus*) embryonic macrophages, represents an established in vitro experimental model for investigating infectious diseases [13,14,15,16], drug repurposing [17], cancer therapy [18], and immune responses [19,20], since this cell line has macrophage-like properties such as nonspecific esterase activity, phagocytic capacity, Fc receptor and CD14 expression [21,22]. In the present study, we propose Fcwf-4 feline macrophages as a meaningful in vitro model to investigate macrophage–*Leishmania* interactions and to evaluate whether exposure to glucuronoxylomannan (GXM), an immunomodulatory capsular polysaccharide produced by *Cryptococcus neoformans*, influences *Leishmania* infection in feline macrophages. The use of *L. amazonensis* species was choice since cases of this species have been reported in Americas [4,23] and is the causative agent of cutaneous and diffuse cutaneous in humans.

Cryptococcosis is an important fungal disease in cats and has also been reported in a variety of wild and exotic animal species [24]. Therefore, investigating the effects of GXM on *Leishmania* infection may provide insights into how pathogen-derived molecules can modulate macrophage susceptibility to parasitic infection. This experimental approach may contribute to a better understanding of the cellular interactions underlying feline Leishmaniasis, particularly in the context of exposure to microbial products.

## 2. Materials and Methods

### 2.1. Feline Macrophages Lineage

The feline macrophage cell line Fcwf-4 was purchased from Banco de células do Rio de Janeiro (BCRJ code 0334) and cultured in DMEM medium (Sigma-Aldrich, St. Louis, MO, USA) supplemented with 10% fetal bovine serum (FBS; Gibco, New York, NY, USA) and maintained at 37 °C/5% CO_2_.

### 2.2. Parasites

*L. amazonensis* promastigotes (MHOM/BR/75/Josefa) were maintained in Schneider’s insect medium (Sigma) supplemented with 10% fetal bovine serum (Gibco) at 27 °C. After 4 days of culture, parasites were washed in PBS (phosphate-buffered saline) by centrifugation at 2000× *g* for 10 min at 22 °C. Cells were resuspended in PBS and counted using a Neubauer chamber (Sigma-Aldrich, St. Louis, MO, USA).

### 2.3. Capsular GXM Purification

Glucuronoxylomannan (GXM) was isolated from the culture supernatant of encapsulated *Cryptococcus neoformans* strains using a protocol adapted from previously described methods [A, B]. Briefly, fungal cells were cultivated in a defined liquid medium at 37 °C under continuous agitation (100 rpm) for 5 days. Following incubation, cells were removed by centrifugation at 12,000× *g* for 1 h at 4 °C. The capsular GXM present in the cell-free supernatant were subsequently recovered by precipitation with three volumes of cold ethanol.

GXM was further purified by differential precipitation using cetyltrimethylammonium bromide (CTAB), as previously described [25]. The ethanol-precipitated polysaccharide fraction was dissolved in 0.2 M NaCl at a concentration of 10 mg/mL. CTAB was gradually added at a ratio of 3 mg per mg of polysaccharide, followed by the addition of 0.05% CTAB solution to promote selective precipitation of GXM. The resulting precipitate was collected by centrifugation and sequentially washed with 2% acetic acid in ethanol and 90% ethanol. The GXM-containing precipitate was then dissolved in 1 M NaCl, followed by the addition of three volumes of ethanol to induce a second precipitation step. After centrifugation, the precipitate was washed again with 2% acetic acid in ethanol and subsequently with ethanol. Finally, the purified GXM was dissolved in distilled water and lyophilized.

To minimize potential lipopolysaccharide (LPS) contamination, the lyophilized GXM preparations were dissolved in LPS-free water and subjected to an additional purification step using a Polymyxin B-agarose column (Sigma-Aldrich, St. Louis, MO, USA) equilibrated with LPS-free water. GXM was eluted with 12 mL of LPS-free water, collected, and lyophilized prior to experimental use.

All GXM preparations were also screened for potential *Mycoplasma* contamination before being used in cell-based experiments. Only preparations that tested negative for Mycoplasma contamination were used in subsequent assays.

### 2.4. Evaluation of Viability of Feline Macrophages Infected with Leishmania spp.

Fcwf-4 feline macrophages were seeded (2 × 10^5^ cells/well) in 96-well plates, and non-adherent cells were removed after 2 h of incubation at 37 °C/5% CO_2_. Cells were then treated or not with 100 µg/mL GXM and incubated for 24 h. Subsequently, cells were infected with *L. amazonensis* promastigotes at a ratio of 10 parasites:1 macrophage and incubated at 37 °C/5% CO_2_ for 2 h. Cells were then washed and further incubated for 24 and 48 h. After incubation, cells were washed with PBS at 37 °C and incubated with 50 µL of XTT solution (Sigma-Aldrich; 0.001 g/mL) containing 10% phenazine methosulfate (PMS, 500 µM). After 4 h at 37 °C, absorbance was measured at 450 nm using a microplate reader. Cell viability (%) was determined relative to untreated, uninfected control cells (100%). TritonX-100 10% was used as negative control.

### 2.5. Infection Kinetics and Determination of Infection Index

Fcwf-4 feline macrophages were plated (2 × 10^5^ cells/well) on 13 mm glass coverslips in 24-well plates. Non-adherent cells were removed after 2 h at 37 °C/5% CO_2_. Cells were treated with GXM [100 µg/mL] for 24 h and then infected with *L. amazonensis* promastigotes (10 parasites:1 macrophage ratio) for 2 h. After washing, cells were incubated for 2, 24, and 48 h. Coverslips were stained using the Giemsa method. The infection index (% infected macrophages × number of parasites per macrophage) was determined by counting at least 200 cells under light microscopy (1000× magnification).

### 2.6. Parasite Load

Fcwf-4 feline macrophages were plated (2 × 10^5^ cells/well) in 24-well plates. After removal of non-adherent cells, macrophages were treated with GXM [100 ng/mL] for 24 h and then infected with *L. amazonensis* promastigotes (10 p:1 m ratio) for 2 h at 37 °C/5% CO_2_. Cells were washed and incubated in DMEM medium for 24 h. Subsequently, cells were washed and Schneider’s insect medium was added, followed by incubation at 27 °C. Released parasites from the cells were counted in the supernatant using a Neubauer chamber on days 3, 5, and 7 post incubation.

### 2.7. Nitric Oxide (NO) Detection

Fcwf-4 feline macrophages (2 × 10^5^ cells/well) were seeded in 96-well plates and treated or not with 100 µg/mL GXM for 24 h at 37 °C/5% CO_2_. Cells were then infected or not with *L. amazonensis* (10:1 ratio). After 2 h, cultures were washed and incubated with fresh medium with or without LPS (400 ng/mL) for 24 h. Supernatants were collected and centrifuged. Samples were transferred to a new plate, and a sodium nitrite standard curve was prepared. Griess reagent (1:1 mixture of solution A: 1% sulfanilamide in 5% phosphoric acid; solution B: 0.1% N-(1-naphthyl)ethylenediamine; Sigma) was added (50 µL). After 15 min, absorbance was measured at 540 nm using a microplate reader (Varioskan LUX, Thermo Fisher Scientific, Waltham, MA, USA).

#### Reactive Oxygen Species (ROS) Detection

Fcwf-4 feline macrophages (2 × 10^5^ cells/well) were plated and treated as described above. After infection and incubation with or without LPS (400 ng/mL) for 24 h, cells were washed and incubated with CM-H2DCFDA probe (10 µM; invitrogen) for 60 min at 37 °C in the dark. Fluorescence intensity was measured using a fluorescence reader (Varioskan LUX) at excitation 493 nm and emission 522 nm.

### 2.8. Statistical Analysis

All data were analyzed using GraphPad Prism 9.5 software. Data normality was assessed using the D’Agostino–Pearson omnibus normality test. Comparisons between two groups were performed using *Student’s t-test*. For comparisons involving three or more groups, one-way ANOVA was used, and for analyses involving two independent variables, two-way ANOVA was applied. Differences were considered statistically significant at * *p* < 0.05.

## 3. Results

To establish the Fcwf-4 feline macrophage cell line as an in vitro model for *Leishmania amazonensis* infection, we first evaluated its ability to interact with and support parasite infection. Fcwf-4 macrophages were pretreated with the capsular polysaccharide glucuronoxylomannan (GXM) or left untreated before incubation with *L. amazonensis* promastigotes for 2, 24, or 48 h. Infection index, was determined, considering both the percentage of infected macrophages and the number of intracellular parasites per infected cell.

**Figure 1:**
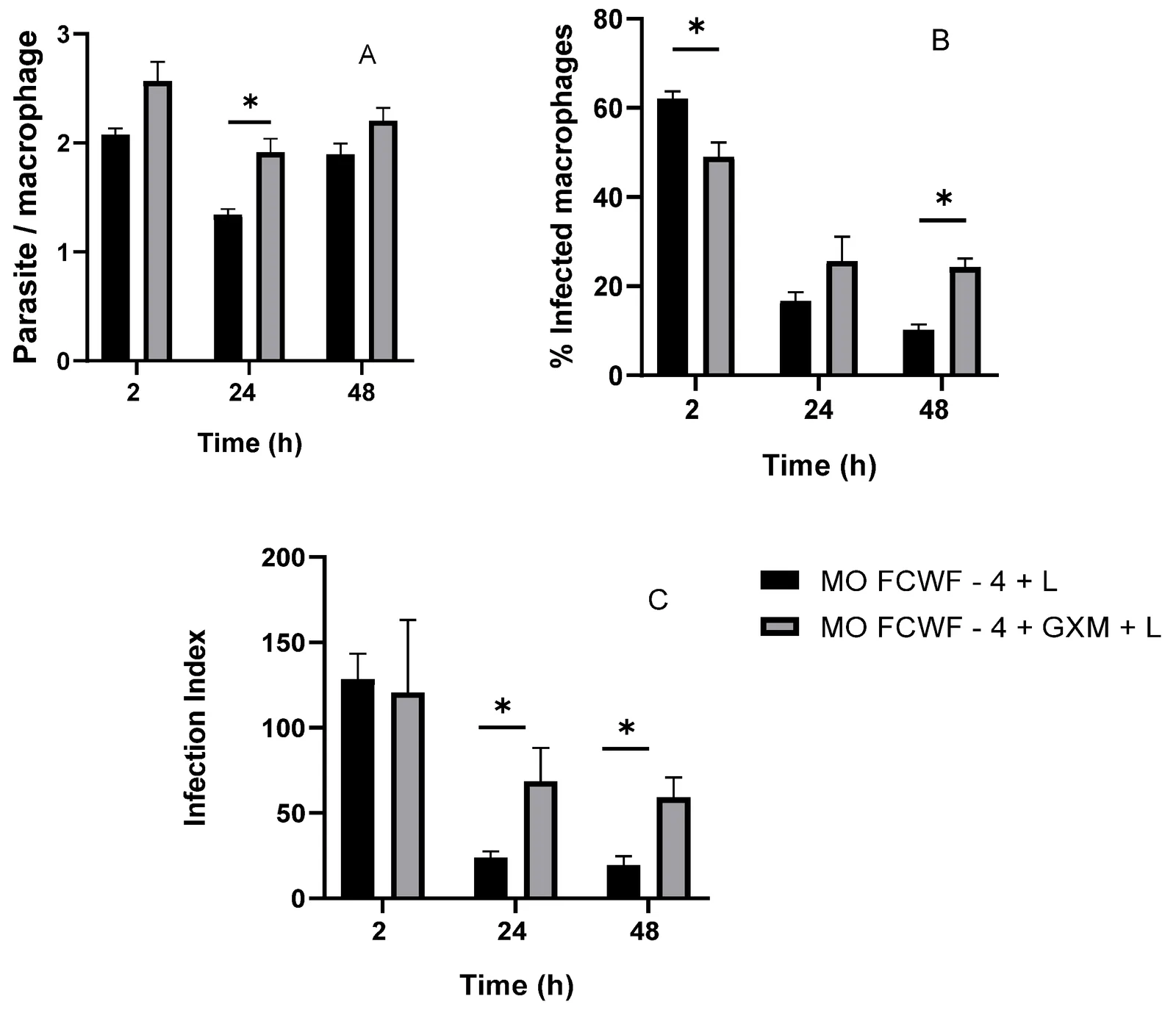
Infection of Fcwf-4 feline macrophages with *Leishmania amazonensis.* Fcwf-4 macrophages were treated or not with GXM [100 µg/mL]) and incubated for 24 h. Following treatment, the cells were infected with *L. amazonensis* promastigotes at a ratio of 10 p:1 m for 2 h at 37 °C/5% CO_2_. Cultures were then washed and maintained for 2, 24, and 48. At the end of each period, the cells were stained with Giemsa, and the infection index was determined by counting at least 200 macrophages per coverslip. (**A**) Number of parasites per infected cell; (**B**) % of infected cells; and (**C**) infection index. Data are expressed as mean ± SD from three independent experiments performed in triplicate. * *p* < 0.05.

Our results demonstrated that Fcwf-4 macrophages efficiently recognized, bound, and internalized *L. amazonensis* promastigotes, confirming that this feline cell line is permissive to parasite infection. Analysis of the parasite per macrophage revealed no significant differences between untreated and GXM-treated macrophages after 2 h of interaction, indicating that GXM pretreatment did not influence the initial parasite uptake (Figure 1A). However, after 24 h of infection, macrophages exposed to GXM displayed a significantly greater parasite burden, with an average of 1.9 intracellular parasites per macrophage compared with 1.3 parasites per macrophage in untreated cells. By 48 h, although intracellular parasites remained detectable, no statistically significant differences were observed in the number of parasites per macrophage between the two groups (Figure 1A).

The percentage of infected macrophages followed a distinct temporal pattern. At the earliest time point (2 h), approximately 62% of untreated macrophages were infected, whereas GXM pretreatment resulted in a lower proportion of infected cells (49%), suggesting that GXM may transiently interfere with the initial parasite-cell interaction (Figure 1B). At 24 h, no significant differences were detected between the experimental groups. In contrast, after 48 h of infection, GXM-treated macrophages exhibited a significantly higher proportion of infected cells (24%) than untreated macrophages (10%), indicating that GXM favors the maintenance or persistence of infection over time (Figure 1B).

**Figure 2:**
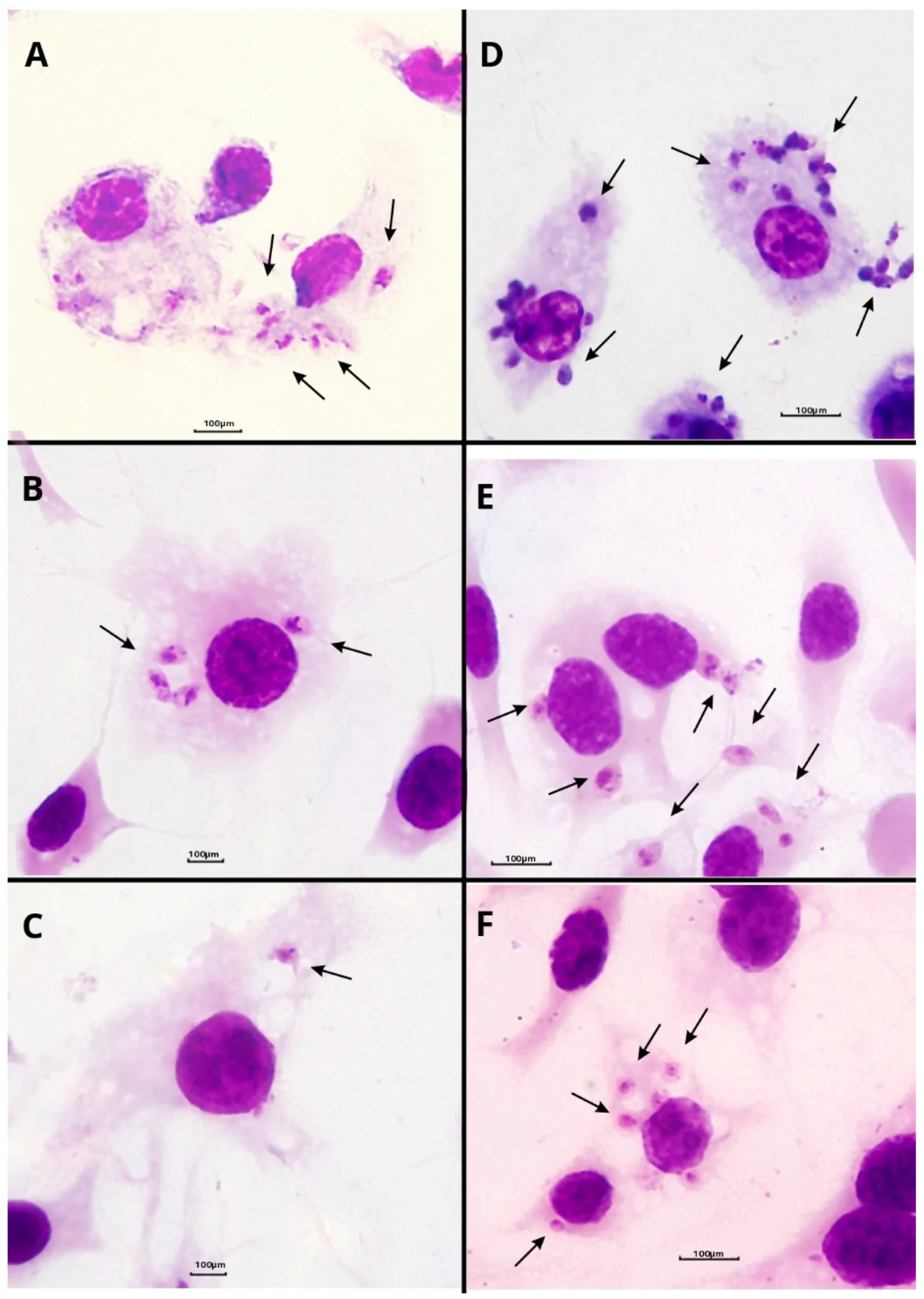
*L. amazonensis* infected-Fcwf-4 feline macrophages at 2 (**A**), 24 (**B**) and 48 h (**C**). GXM treated-*L. amazonenis* infected Fcwf-4 feline macrophages at 2 (**D**), 24 (**E**) and 48 h (**F**). Arrows indicated parasites. Bars = 100 µM.

**Figure 3:**
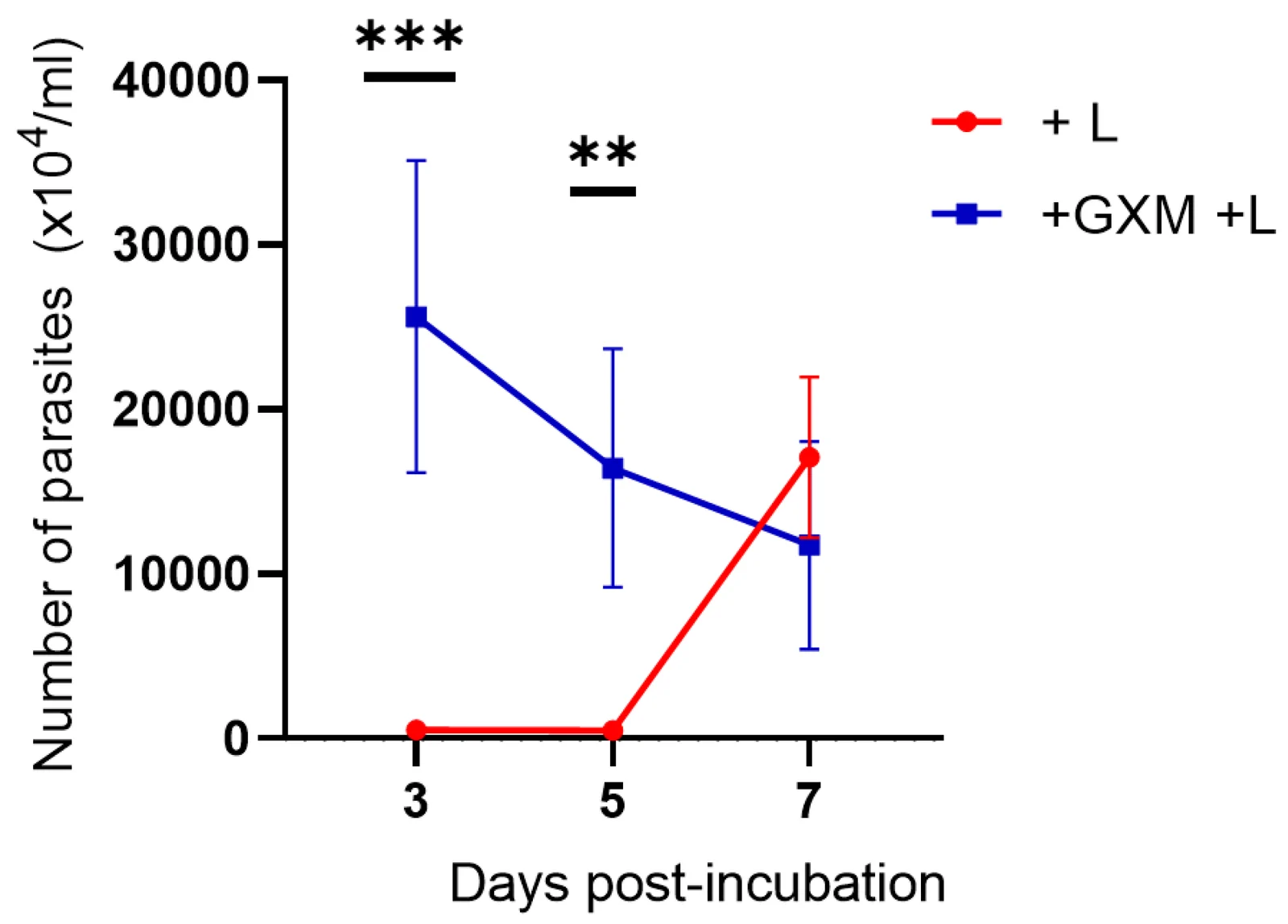
Analysis of parasite load in Fcwf-4 feline macrophages. Feline macrophages were treated or not with GXM [100 µg/mL] for 24 h at 37 °C/5% CO_2_. Then, cultures were infected with *L.a*. promastigotes at a ratio of 10 p:1 m and incubated for 2 h under the same conditions. After that, cells were washed with PBS and maintained for 24 h as described above. Thereafter, the culture medium was replaced with Schneider’s medium, and infected macrophages were incubated in a at 27 °C. Parasite load was quantified by counting released promastigotes in a Neubauer chamber at 3, 5, and 7 days post-incubations. The graph is a representative of 3 experiments performed in triplicate. ** *p* = 0.004; *** *p* < 0.001.

When both parameters were integrated into the infection index, GXM pretreatment markedly enhanced the susceptibility of Fcwf-4 macrophages to *L. amazonensis*. The infection index was 2.9-fold higher at 24 h and 3.04-fold higher at 48 h in GXM-treated macrophages compared with untreated controls (Figure 1C). Representative optical microscopy images corroborated these quantitative findings, showing a visibly greater intracellular parasite load in GXM-treated macrophages than in untreated cells (Figure 2). The parasite load analyses inside feline macrophages showed that the number of viable parasites released from GXM-treated cells were higher compared to untreated feline macrophages (Figure 3).

To exclude the possibility that the increased parasite burden resulted from cytotoxic effects of GXM or parasite infection, macrophage viability was evaluated under all experimental conditions. Cell viability remained consistently high throughout the experimental period, with approximately 98% of cells remaining viable after both 24 and 48 h of incubation, regardless of GXM treatment or *L. amazonensis* infection (Figure 3). These findings indicate that neither GXM nor parasite infection compromised macrophage viability, demonstrating that the enhanced infection observed in GXM-treated cells cannot be attributed to differences in cell survival.

**Figure 4:**
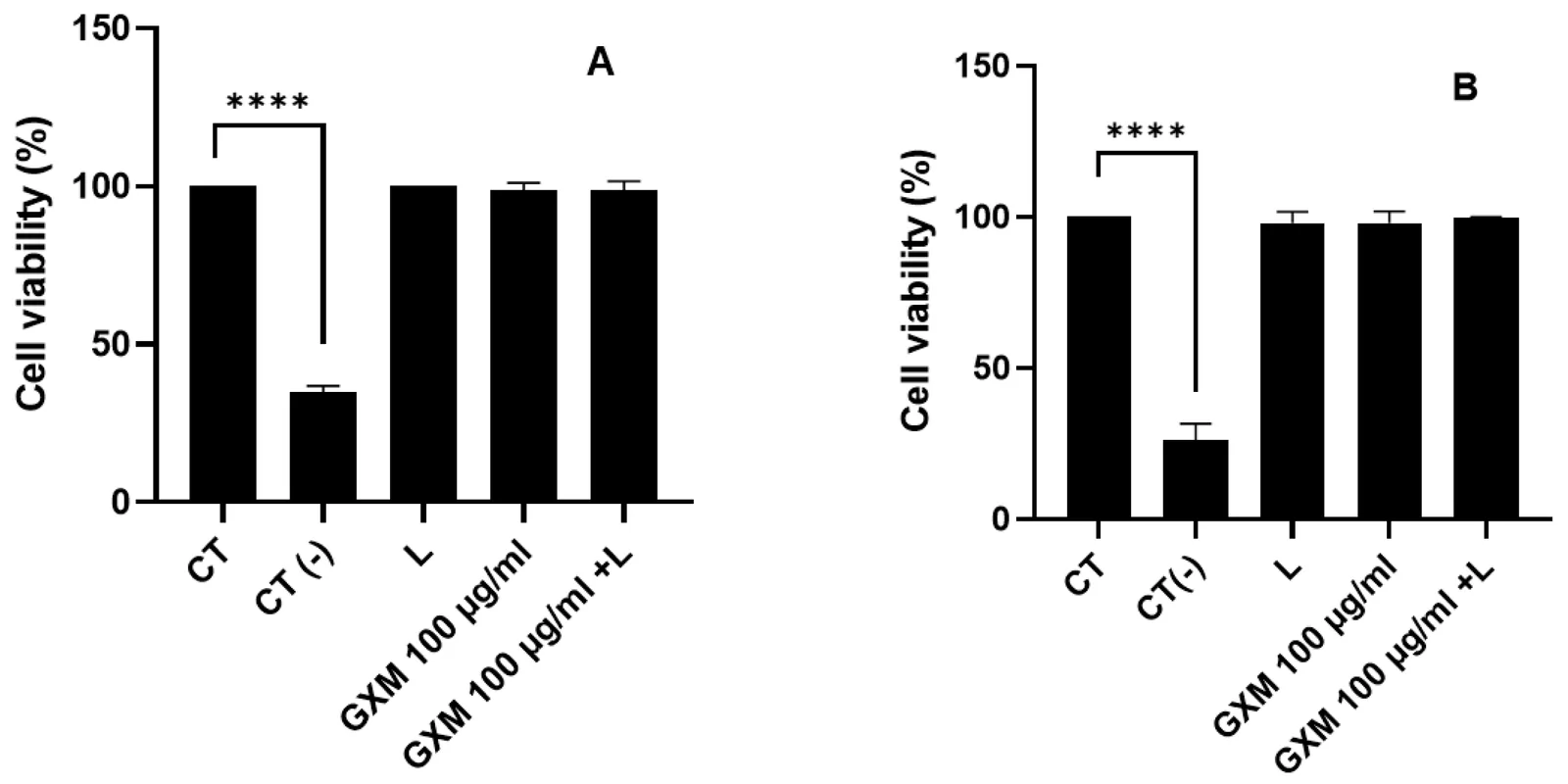
Fcwf-4 feline macrophages Viability. Fcwf-4 macrophages were treated or not glucuronoxylomannan (GXM; 100 µg/mL) and incubated for 24 h. Then, the cells were infected with L. a. promastigotes at a ratio of 10 p:1 m for 2 h at 37 °C/5% CO_2_. After infection, cultures were washed and incubated for (**A**) 24 h or (**B**) 48 h under the same conditions. Cell viability was assessed at each time point relative to untreated and uninfected control cells. Data are expressed as mean ± SD from three independent experiments in triplicate. CT, control; L.a., *L. amazonensis*; GXM, glucuronoxylomannan, CT(-) triton 10%. Data are expressed as mean ± SD from three independent experiments performed in triplicate. **** *p* < 0.0001.

Because nitric oxide (NO) is a major macrophage effector molecule involved in the control of intracellular pathogens, we next investigated whether GXM modulated NO production in Fcwf-4 macrophages infected with *L. amazonensis*. Macrophages were cultured in the presence or absence of GXM, infected or not with *L. amazonensis*, and stimulated or not with lipopolysaccharide (LPS). As expected, LPS stimulation induced a robust activation of Fcwf-4 macrophages, resulting in a 3.1-fold increase in nitrite production compared with unstimulated control cells (Figure 4). Neither GXM treatment alone nor *L. amazonensis* infection alone significantly altered basal NO production relative to untreated controls. However, *L. amazonensis* markedly suppressed the NO response induced by LPS stimulation, reducing nitrite production by approximately 3.47-fold. (Figure 4).

**Figure 5:**
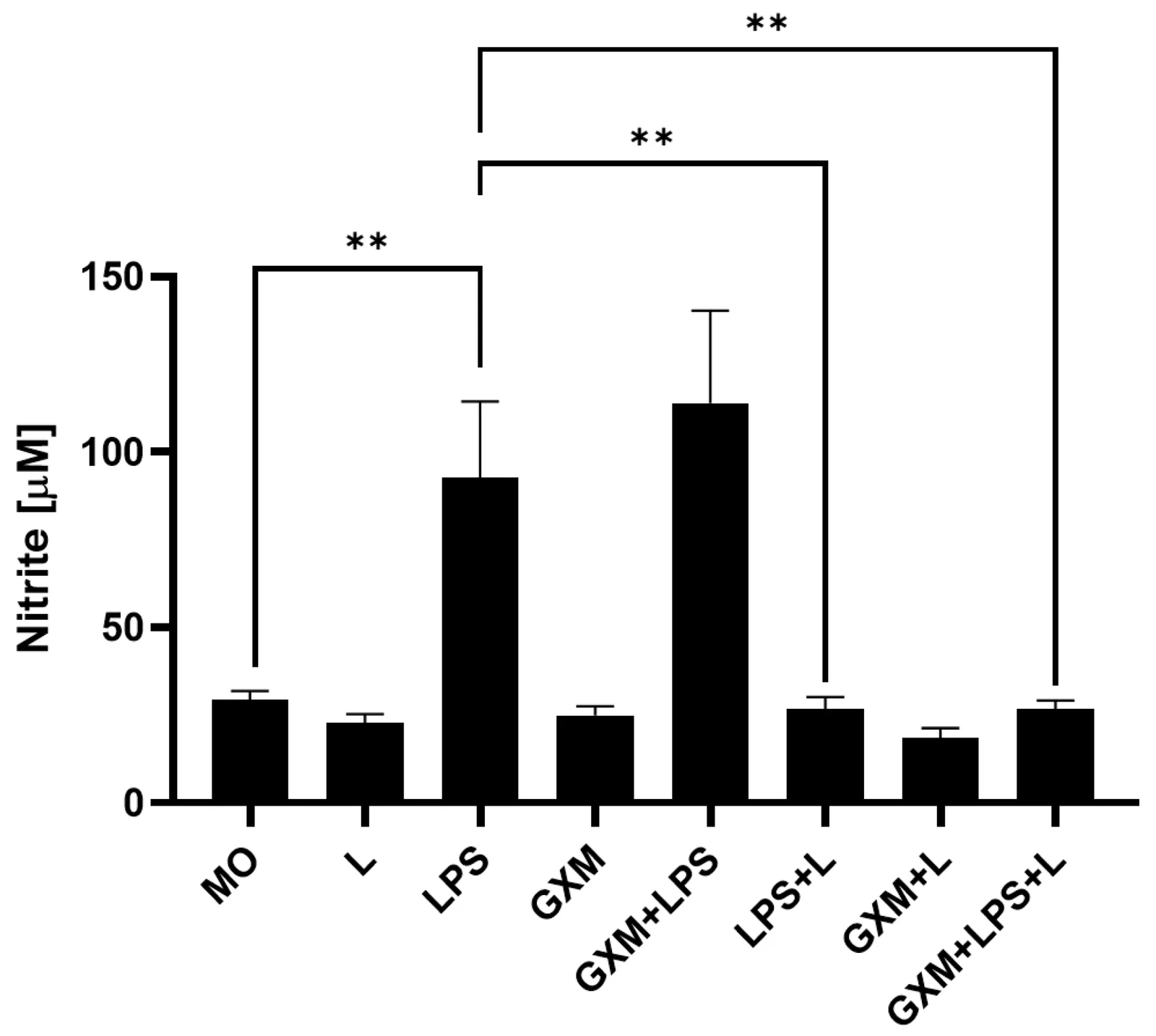
Nitric Oxide (NO) production by Fcwf-4 feline macrophages treated with GXM and/or infected with *L. amazonensis*. Fcwf-4 feline macrophages were treated or not with GXM [100 µg/mL] for 24 h at 37 °C/5% CO_2_. Then, the cells were infected or not with *L. amazonenis* (10 p:1 m). After 2 h of infection, LPS [400 ng/mL] was added or not. The cultures were maintained for an additional 24 h at 37 °C/5% CO_2_. Nitrite concentration was determined using Griess reagent. L = *L. amazonensis*, GXM = glucuronoxylomannan and LPS = lipopolysaccharide. Data + SD from 4 independent experiments in quadruplicate. ** *p* = 0.005.

**Figure 6:**
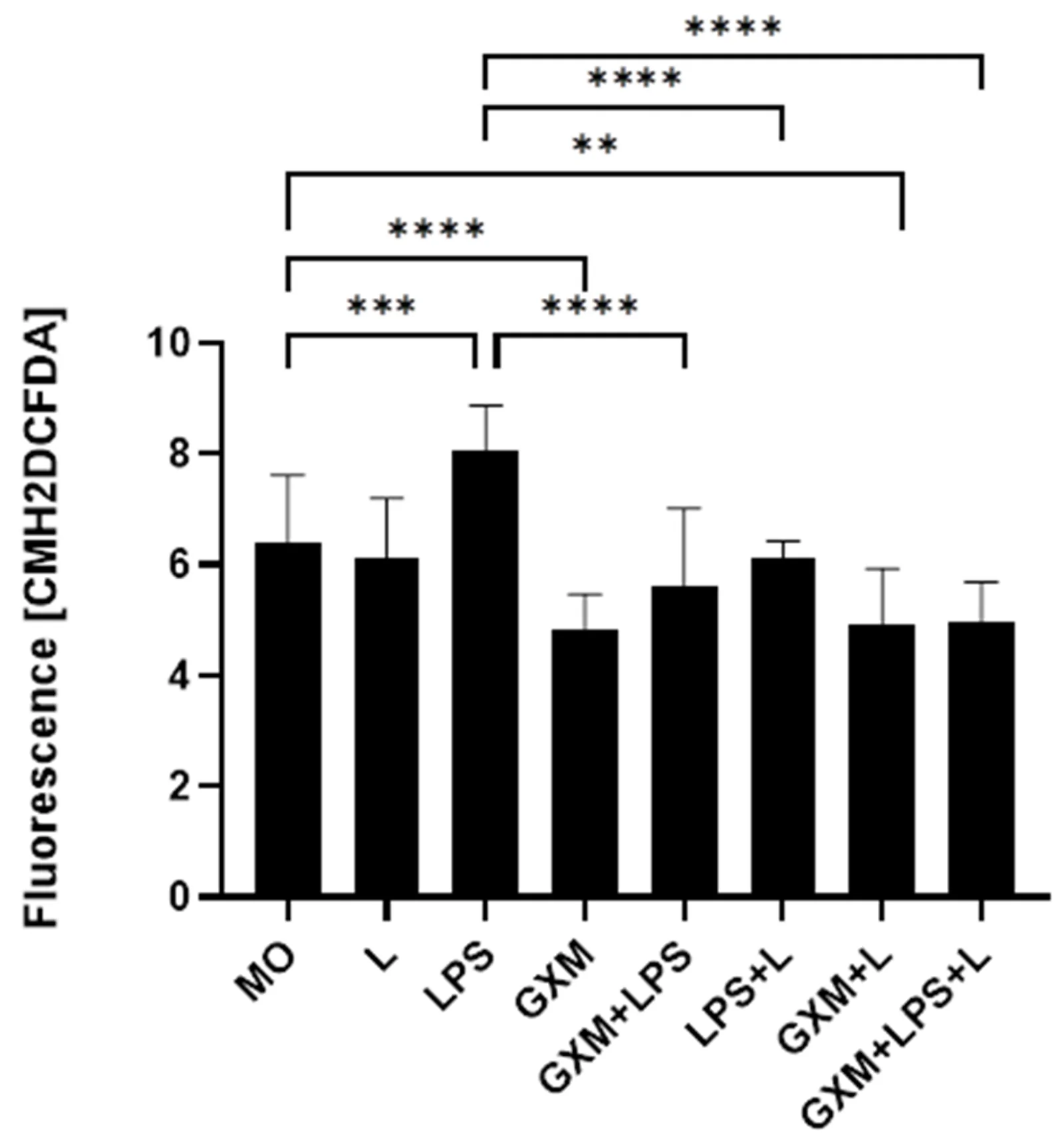
Reactive species of oxygen (ROS) production by Fcwf-4 feline macrophages treated with GXM and/or infected with *L. amazonensis.* Fcwf-4 feline macrophages were treated or not with GXM [100 µg/mL] for 24 h at 37 °C/5% CO_2_. Then, the cells were infected or not with *L. amazonenis* (10 p:1 m). After 2 h of infection, LPS [400 ng/mL] was added or not. The cultures were maintained for an additional 24 h at 37 °C/5% CO_2_. At the end of the incubation period, the cells were incubated with CM-H2DCFDA probe for 60 min in the dark at 37 °C. The mean fluorescence intensity was read using excitation at 493 nm and emission at 522 nm. L = *L. amazonensis*, GXM = glucuronoxylomannan and LPS = lipopolysaccharide Data = Mean ± SEM from three experiments performed in quadruplicate. ** *p* = 0.004; *** *p* < 0.001; **** *p* < 0.0001.

Reactive oxygen species (ROS), another critical component of the macrophage antimicrobial response, were also evaluated. LPS stimulation significantly increased ROS generation by approximately 1.25-fold compared with unstimulated macrophages, confirming the responsiveness of Fcwf-4 cells to inflammatory activation (Figure 5). In contrast, GXM treatment significantly reduced basal ROS production by approximately 1.3-fold, indicating that this cryptococcal polysaccharide exerts an inhibitory effect on macrophage oxidative metabolism. Infection with *L. amazonensis* alone did not significantly alter ROS production relative to uninfected control macrophages. Nevertheless, under LPS-stimulated conditions, GXM pretreatment significantly attenuated the oxidative burst, reducing ROS production by approximately 1.6-fold compared with untreated LPS-stimulated macrophages (Figure 5). Taking together, our findings demonstrate that Fcwf-4 feline macrophages are permissive to *Leishmania amazonensis* infection and that the parasite actively modulates macrophage effector responses, particularly by suppressing nitric oxide (NO) production following LPS stimulation. Importantly, exposure to glucuronoxylomannan (GXM), an immunomodulatory polysaccharide of the *C neoformans* capsule, further enhanced *Leishmania* survival and inhibited reactive oxygen species (ROS) production under resting, LPS-stimulated, and *L. amazonensis*-infected conditions. These findings reveal a potential mechanism by which fungal-derived immunomodulatory molecules can alter macrophage antimicrobial responses and promote the persistence of an intracellular parasite. More broadly, our results highlight the potential relevance of GXM-mediated immunomodulation in shaping host–pathogen interactions and provide new insights into how pathogen-derived molecules may influence immune responses beyond their original infectious context. (Figure 6).

## 4. Discussion

Studies addressing the feline macrophage response to *Leishmania* infection remain limited, restricting our understanding of the cellular mechanisms underlying host–parasite interactions in this species. The establishment of an experimental model based on the Fcwf-4 cell line provides a controlled and reproducible system to investigate early events in feline macrophage–*Leishmania* interactions and to assess mechanisms underlying parasite persistence and immune modulation. Fcwf-4 cells have previously been used in several experimental contexts, including infectious disease models [13,14,15,16], and studies of immune responses [19,20]. Thus, the use of this feline macrophage cell line may provide a valuable platform for investigating host–parasite interactions while reducing the need for animal experimentation during the initial stages of experimental studies.

Our results demonstrate that Fcwf-4 cells are susceptible to infection with *L. amazonensis*, with intracellular parasites detected at all evaluated time points (2, 24, and 48 h). Interestingly, the infection index decreased over time, suggesting that Fcwf-4 macrophages can partially control parasite burden without complete eliminating the infection. These findings are consistent with recent observations showing that feline monocyte-derived macrophages can sustain infection with *L. infantum*, the *Leishmania* species most frequently associated with feline infections, as well as with *L. tarentolae*, a species primarily associated with reptiles [26]. Similarly, feline blood monocyte-derived macrophages have been shown to support *L. infantum* infection in vitro for up to 72 h [11]. However, the reduction in *L. amazonensis* infection observed in Fcwf-4 cells was not reported in *L. infantum*-infected monocyte-*derived* macrophages. Studies using canine and murine macrophage cell lines show that *L. amazonensis* often exhibits rapid early survival reduction (around 40% at 24 h), whereas *L. infantum* can display higher relative persistence depending on the host macrophage origin [27]. Proteomic analysis in murine peritoneal macrophages infected with *L. infantum* and *L. amazonensis* identified differential protein abundant, which can affect infection course [28]. These differences may reflect, at least in part, the distinct biological characteristics of the *Leishmania* species and macrophage models used in each study. Although *L. amazonensis*, a cutaneous species, is not the main species identified in cats, cases of *L. amazonesnsis, L. braziliensis and L. mexicana* have been reported in New World [4,23,29], the use of these species can contribute to understand macrophages species-specific response. Collectively, these findings indicate that Fcwf-4 macrophages can support intracellular *L. amazonensis* infection while retaining the capacity to partially restrict parasite replication or persistence over time.

Feline cryptococcosis is an important systemic fungal infection, and cases of *Cryptococcus* spp. and *Leishmania* co-infection have been reported in cats [10,21]. Glucuronoxylomannan (GXM), the major capsular polysaccharide of *Cryptococcus neoformans*, has well-established immunomodulatory properties and can alter innate immune responses in different experimental models [30,31,32]. In the present study, GXM treatment increased the infection index in *L. amazonensis*-infected Fcwf-4 macrophages compared with untreated infected cells. A similar effect was recently reported in murine macrophages infected with *L. major* following GXM exposure [31], suggesting that the ability of GXM to promote *Leishmania* persistence may extend across different macrophage species and *Leishmania* models.

The immunomodulatory activity of GXM has been associated with several mechanisms, including induction of IL-10 production, modulation of innate immune responses, signaling through Fcγ receptor II (FcγRII), and TGF-beta production, which can activate inhibitory pathways [30,32]. These effects may contribute to the increased infection index observed in GXM-treated Fcwf-4 macrophages by impairing macrophage antimicrobial functions. Importantly, the increased infection index was not associated with reduced cell viability. Fcwf-4 cell viability was maintained under all experimental conditions, consistent with previous findings showing that GXM is not cytotoxic to murine peritoneal macrophages at concentrations of up to 300 µg/mL following 24 h of exposure [31]. Therefore, the increased infection index observed following GXM treatment is unlikely to be attributable to host–cell death and instead supports a role for GXM-mediated immunomodulation in facilitating intracellular parasite persistence.

To investigate mechanisms potentially contributing to the partial control of *L. amazonensis* infection by Fcwf-4 macrophages, we evaluated the production of reactive nitrogen and oxygen species. Fcwf-4 macrophages produced NO following stimulation with 400 ng/mL LPS, although this concentration is relatively high compared with concentrations commonly used to stimulate murine macrophage cell lines such as RAW 264.7. Notably, *L. amazonensis* infection reduced NO production in LPS-stimulated J774-G8murine macrophages without decreased iNOS mRNA expression, suggesting that the parasite affects post-transcriptional events of NO synthesis [33]. It is necessary further investigation to evaluate if it is the case of feline macrophages. Furthermore, murine peritoneal macrophages infected with *L. major* and treated with GXM did not exhibit a significant increase in NO production [31]. In the present study, *L. amazonensis* infection reduced NO production in feline macrophages, the same effect was not observed by GXM treated cells. Since GXM immunomodulatory action in the murine model was interpreted in the context of M1/M2 polarization [31] it cannot be assumed that the same mechanism, or even the same effect, will occur in feline macrophages infected with *L. amazonensis*. Because reactive nitrogen species contribute to macrophage-mediated control of intracellular pathogens, the suppression of NO production may create a cellular environment that favors *Leishmania* persistence.

ROS production showed a similar pattern of immunomodulation. LPS stimulation increased ROS production by Fcwf-4 macrophages, whereas GXM treatment reduced ROS levels compared with untreated cells. This observation is consistent with findings in canine DH82 macrophages, in which LPS and IFN-γ increased ROS production, while GXM significantly reduced ROS levels under the same stimulatory conditions [34]. Notably, GXM also reduced basal ROS production in unstimulated Fcwf-4 macrophages, further supporting its broad inhibitory effect on macrophage activation. In contrast, *L. amazonensis* infection alone did not significantly alter ROS production compared with unstimulated macrophages. Similarly in murine macrophages from CBA mice showed that *L. amazonensis* did not induce ROS production compared to *L. major* [35]. Recent evidence indicates that ROS production is not merely a terminal antimicrobial mechanism but also participates in early inflammatory signaling and metabolic adaptation, thereby influencing cytokine production and antimicrobial responses [36]. Therefore, the reduction in ROS induced by GXM, including under basal conditions, may interfere with early macrophage activation and metabolic adaptation, potentially contributing to an intracellular environment permissive for *Leishmania* persistence. This possibility warrants further investigation.

**Figure 7:**
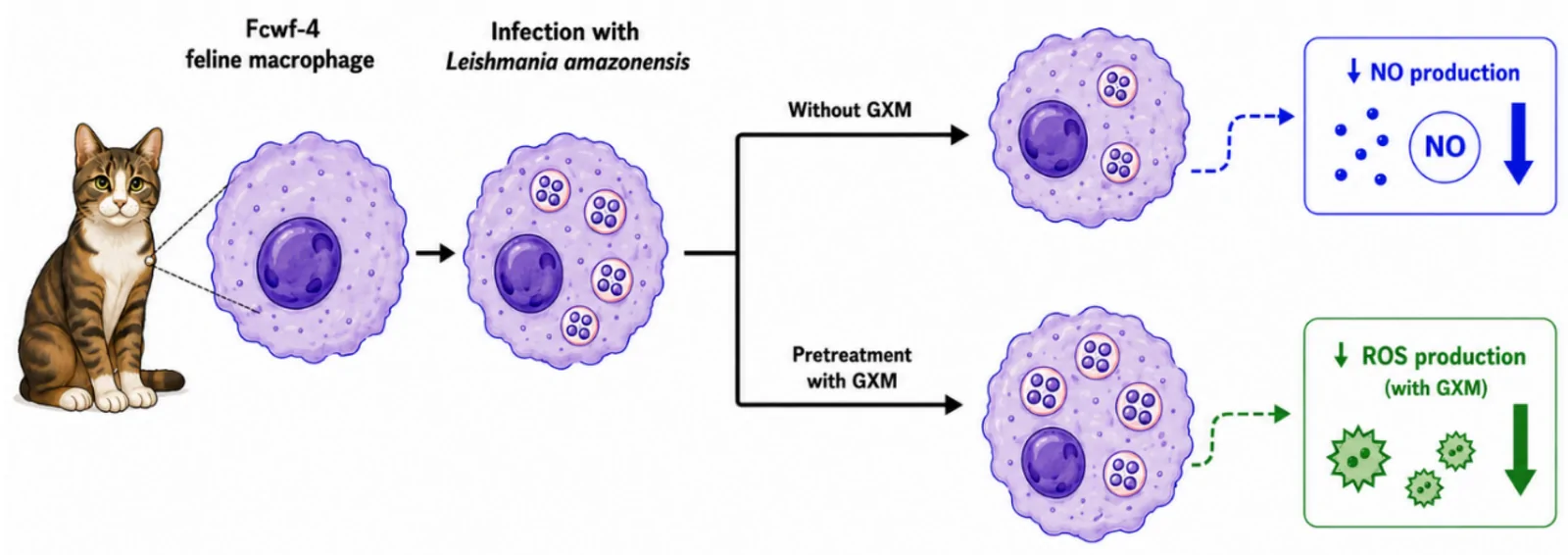
GXM-mediated modulation of *Leishmania amazonensis* infection and macrophage oxidative responses in feline macrophages. Fcwf-4 feline macrophages interact with and support infection by *Leishmania amazonensis*. *L. amazonensis* infection inhibits nitric oxide (NO) production by LPS-stimulated feline macrophages. The presence of glucuronoxylomannan (GXM), an immunomodulatory polysaccharide from the *Cryptococcus neoformans* capsule, enhances *Leishmania* survival within macrophages. GXM also suppresses reactive oxygen species (ROS) production by resting, LPS-stimulated, and/or *L. amazonensis*-infected Fcwf-4 macrophages, indicating that GXM promotes an immunoregulatory macrophage environment that favors parasite persistence.

The ability of feline macrophages to partially control *Leishmania* infection may also be relevant to the differences in susceptibility observed between cats and dogs. Cats are generally considered less susceptible to clinical Leishmaniasis than dogs, although persistent parasitemia can occur [3]. Comparative studies in endemic areas have suggested that cats and dogs infected with *L. infantum* share several features of immunopathogenesis, although dogs may develop stronger antibody responses and more pronounced parasite-specific immune responses than cats [9]. In naturally infected cats, increased serum levels of IL-12 and IFN-γ have been reported compared with healthy animals [12], while feline blood monocyte-derived macrophages infected with *L. infantum* exhibit increased expression of proinflammatory cytokines [11]. Together, these observations are compatible with the hypothesis that feline macrophages can mount an effective, although potentially incomplete, pro-inflammatory response to *Leishmania*. However, whether this response directly contributes to the lower frequency or severity of clinical disease in cats remains to be established. Importantly, concomitant infections or exposure to immunomodulatory microbial components such as GXM could alter this balance and potentially increase susceptibility to *Leishmania* persistence (Figure 7). Cats are naturally exposed to both *Leishmania* spp. and to co-infections/immunomodulatory agents in endemic areas, and a validated a model of feline macrophage response would fill a specific methodological gap, since most in vitro *Leishmania*–macrophage has been done in murine or human systems. Thus, coinfection or the modulatory effects of products secreted by other microorganisms may represent an important factor influencing the outcome of feline leishmaniasis and deserves further investigation, including using primary macrophages.

## 5. Conclusions

Overall, our findings establish Fcwf-4 feline macrophages as a useful in vitro model for investigating early cellular events during *Leishmania* infection and for dissecting mechanisms of parasite persistence in feline macrophages. Fcwf-4 cells supported infection with *L. amazonensis* promastigotes while retaining the capacity to partially restrict the infection over time. Importantly, exposure to GXM increased the infection index without compromising macrophage viability and was associated with reduced NO and ROS production. These findings provide experimental evidence that fungal-derived immunomodulatory molecules may influence the outcome of *Leishmania* infection in feline macrophages and highlight a potential mechanism by which co-infections or exposure to products derived from other microorganisms could modify host–parasite interactions. The Fcwf-4 model may therefore provide a useful platform for future studies aimed at defining the molecular pathways in feline macrophage activation, parasite persistence, and the interplay between *Leishmania* and other pathogens.

## Data Availability

The original contributions presented in this study are included in the article. Further inquiries can be directed to the corresponding author.
